# A nurse practitioner model for the assessment of suspected prostate cancer referrals is safe, cost and time efficient

**DOI:** 10.3332/ecancer.2019.994

**Published:** 2019-12-18

**Authors:** Lawrence Drudge-Coates, Vitra Khati, Randolph Ballesteros, Clarissa Martyn-Hemphill, Christian Brown, James Green, Ben Challacombe, Gordon Muir

**Affiliations:** 1 Department of Urology, King’s College Hospital NHS Foundation Trust, SE5 9RS London, UK; 2 Department of Urology, Barts Health NHS Trust, London EC1A 7BE, UK; 3 Department of Urology, Guy’s and St Thomas’ NHS Foundation Trust, London SE1 9RT, UK

**Keywords:** nurse led, prostate cancer, cancer referral, cost-effectiveness, patient safety

## Abstract

**Purpose:**

To evaluate the outcomes from a Urology Nurse Practitioner (UNP)-led service for the initial assessment and diagnostic decision making and for suspected prostate cancer referrals.

**Methods:**

Using a modified Delphi analysis approach, a panel of Urological Prostate Cancer specialists were asked to review the UNP management plans of a convenience sample of 60 randomly selected patient cases – between June 2012 and June 2015. The panel was required to establish consensus or identify divergence of clinical practice, based on five key statements. In addition, cost analysis, waiting time and patient satisfaction evaluation were made regarding the nurse-led service.

**Results:**

In 87% (52/60 cases), consensus was reached by the panel that the UNP management plan was entirely appropriate and in only two cases was there discordance, where the panel felt that the management plan by the UNP was inappropriate with errors potentially and significantly affecting the patient. Over the 3 years, a modest cost saving of £11,500.38 was realised, which due to increased referrals has now realised in 1 year (2017/18) a saving of £11,335.50. Compared to the previous physician-led service, waiting times for patient appointment fell by 52% over the 3-year period; 57/63 (90%) patients reported being satisfied with seeing a UNP instead of a doctor for their first appointment; 60/63 (95%) reported that, following the initial hospital visit with the UNP, they had a clear understanding of what the next steps were in their assessment. Overall, 54/63 (86%) were ‘very satisfied’ with the UNP-led service.

**Conclusion:**

Our study demonstrates that a UNP approach to the assessment and management of suspected prostate cancer referrals provides an effective approach to care in an ever-demanding healthcare arena.

Through a supported training programme, urology nurses can deliver a high standard of service.

## Introduction

Accounting for almost 13% of all new cancer cases, prostate cancer is the second most common cancer in the UK. There are around 47,700 new prostate cancer cases in the UK every year, averaging about 130 every day (2014–16) [1]. Since the introduction of the Department of Health guidelines in 2000 [2], all urology departments have been required to see all suspected cancer referrals within 2 weeks, which has led to significant resource implications nationally. In a rapidly changing healthcare landscape where the prevalence of cancer is forecast to increase, financial pressures to deliver cost-effective and timely healthcare, while maintaining high clinical standards, will only continue to increase [3].

Urgent attention, therefore, needs to be directed towards strategic workforce planning. It is essential that we develop a workforce that is both sustainable and cost-effective, which has the appropriate clinical expertise and is multi-professional, in order to meet the growing complex needs of cancer patients [3]. The utilisation of highly skilled and competent Urology Nurse Practitioners (UNPs) has already demonstrated that nurse-led approaches can deliver safe and clinically effective aspects of care, with high levels of patient satisfaction and improved patient outcomes [4–7]. However, to date, no published studies have presented the outcomes of a nurse-led outpatient clinic assessment approach for suspected prostate cancer referrals.

The primary goal of this study was, therefore, to evaluate the outcomes from a UNP-led service, from initial assessment to diagnostic decision making, for suspected prostate cancer referrals. In addition, evaluate waiting times, cost implications and patient recorded outcome measures.

## Patients and methods

A standardised protocol-driven assessment model was devised and agreed in 2012 for suspected prostate cancer referrals.

The initial patient referral by the general practitioner (GP) was based on elevated prostate-specific antigen (PSA) and/or abnormal digital rectal examination (DRE). Following initial triage of the referrals, patients were allocated appointments in one of four weekly nurse-led clinics.

The assessment protocol required all referrals to undergo the following:

- Full medical history.
- DRE.
- Blood tests (including PSA).
- Assessment of lower urinary tract symptoms (LUTS) using the International Prostate Symptom Score (IPSS) assessment tool and uroflowmetry.
- Sexual function assessment: Sexual Health Inventory for Men (SHIM) or International Index of Erectile Function 5 item version (IIEF-5).

Clinical assessment was undertaken on the same day, facilitating a ‘one-stop’ approach. The competencies for the UNP undertaking DREs and medical history had been previously assessed and achieved prior to this model being instigated, using an existing locally agreed cancer network policy and competency framework.

Based on the initial clinical findings, referrals were then stratified by the UNP to No, Low, Intermediate or High risk of prostate cancer, from which the need for pre-biopsy multiparametric Magnetic Resonance Imaging (mpMRI) was determined. Such an approach was adopted to help avoid unnecessary prostate biopsies and improve diagnostic accuracy. This approach was later confirmed in the findings of the Prostate MRI Imaging Study (PROMIS) as highly effective, with the use of mpMRI as a triage and staging test, reducing the need for primary prostate biopsy in patients by 27% [8].

**Figure 1.**
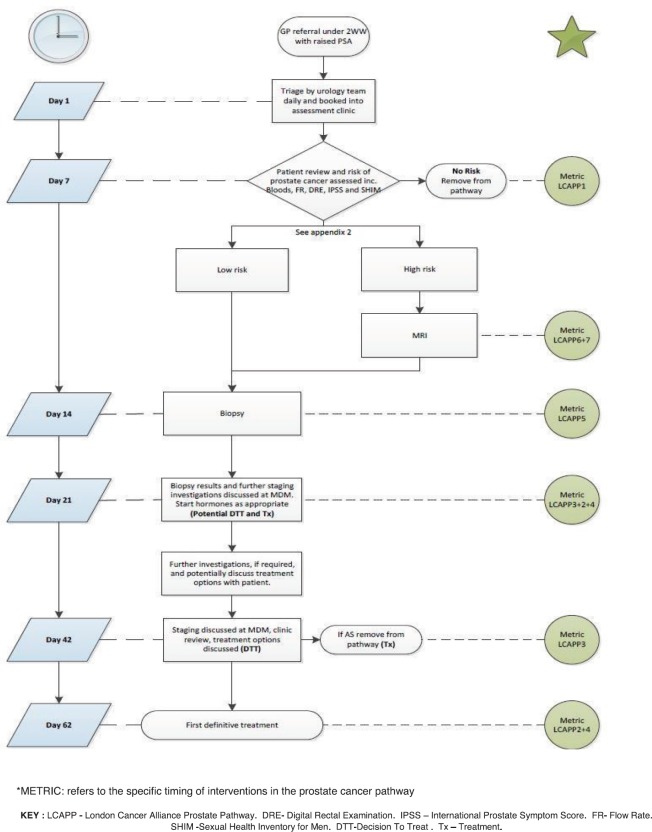
UNP best practice prostate pathway flow diagram and anticipated timescale.

Based on the evaluation of the MRI findings, the need for prostate biopsies was determined and requested, once discussed with the patient, using either a transrectal or transperineal approach (our standard approach changed to primary transperineal biopsy during the course of this study). The nurse-led referral to diagnostic approach is outlined in Figure 1.

This pathway was later adopted as the London Cancer Alliance Best Practice Prostate Pathway in phase 1, with later modification in phase two of the pathway design [9].

## Primary outcomes

To evaluate the outcomes of the service, a modified Delphi analysis technique was employed. Using an initial convenience sample, 60 patient cases (approx. 10%) referred and assessed between June 2012 and June 2015 were randomly selected by a senior member of the clerical staff from the clinic lists. This approach was adopted because it was less time-intensive. In acknowledging its limitations, specific inclusion criteria for the Delphi analysis were enforced. The patient cases were deemed eligible for inclusion, only if all assessments had been completed and a management plan had been outlined.

Three Prostate Cancer specialists (consultant urologists) from local urological cancer centres in London formed the Delphi panel. Each were given clinical summaries of the 60 patients, and independently, without conferring, asked to give their opinion on each management plan generated by the UNP, up to and including the need for prostate biopsies. The Delphi analysis required the urologists to establish consensus or identify divergence of clinical practice and was based on their responses to five key statements (Table 1). This approach was designed to offset the bias of conventional approaches that have been noted in the literature through the pooling of opinions, commonly obtained from group interaction, from dominant individuals or because of conformity that might be sought because of group pressure [10]. Consensus opinion for concordance was reached when all three urologists agreed that the management plan was entirely appropriate.

For divergent opinions, the case was represented once to the Delphi panel for discussion to see if agreement on management could be reached.

## Secondary outcomes

The cost analysis was based on UK NHS prices for a 30-minute clinic appointment, by a mid-band 7 urology nurse (inclusive of London weighting). This was compared to that of a staff/middle-grade urologist, Specialist Training Registrar (ST3/4) and Consultant Urologist, with the inclusion of an additional 50% cost of a nursing assistant, supporting these clinics.

The potential cost-saving associated with a reduction in follow-up appointments was also included, through comparison of the old service pathway to the new nurse-led service. Data related to costs were provided by the business intelligence unit from the first author’s hospital.

Using a convenience sample to evaluate the service, questionnaires were sent out to the first 100 patients, within 30 days of completing the initial UNP assessment and diagnostic pathway.

Waiting times from the point of GP referral to initial clinic assessment were analysed by comparing the previous service led by a urologist up until June 2011 and then the nurse-led service from 2012 to 2015.

This study was registered as an audit at King’s College Hospital and adhered to the standard required by the Health Quality Improvement Partnership (HQIP) [11].

## Results

A total of 558 men were seen in the UNP-led clinic following the referral by their GPs for suspected prostate cancer, from June 2012 to June 2015; the median age was 74 years (range: 39–90 years). After initial assessment, 279 men (50%) underwent further assessment, of which 25 (4.4%) were diagnosed with metastases at presentation and commenced on hormone therapy without a biopsy. Seventeen (3%) were clinically malignant and commenced on watchful waiting. Two non-metastatic patients with very high PSA levels but multiple co-morbidities were also commenced on hormones. A total of 12 (2.2%) men declined prostate biopsies against the medical recommendation.

Further to discussing the results of the pre-biopsy mpMRI, 223/558 men (40%) underwent prostate biopsies, of which 150/223 (67.3%) were diagnosed with prostate cancer; the treatment approaches for these men are outlined in Table 2.

Out of the remaining 279 men, 46/279 (16.5%) underwent mpMRI only, findings of which were categorised by our radiologists based on the Prostate Imaging-Reporting and Data System, version 2 (PI-RADS v2) and in addition included PSA density levels [12]. In 40/46 (87%), results were not suggestive of clinically significant prostate cancer (PIRADS 1 and 2) and hence did not undergo prostate biopsies at that time. In the six (13%) patients who had PIRADS 3 (equivocal regarding the likelihood of clinically significant prostate cancer), three declined a prostate biopsy following the initial mpMRI, two had lesions in the range of 4–6 mm in prostate glands > 100 cc and the remaining patients were undergoing cardiac treatment and were initiated for anticoagulation therapy – all these individuals were, however, followed up in the clinic with continuous review.

**Figure 2.**
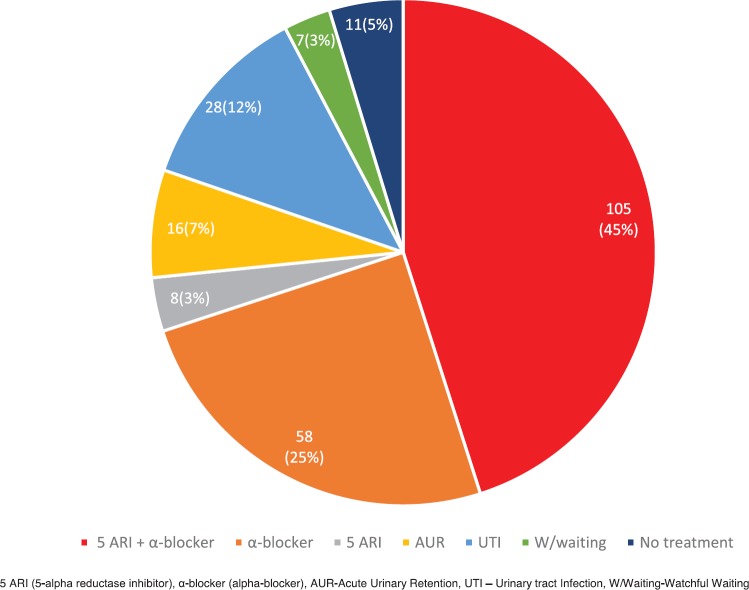
Initial clinical outcomes for those not undergoing further diagnostic investigations (*n* = 233).

A total of 233/279 (83.5%) men did not undergo any further investigations following their initial appointment. These included men who were assessed as clinically benign but warranted treatment for their LUTS (Figure 2). Importantly, all patients undergoing treatment were then subsequently followed up in an outpatient clinic and discharged back to their GPs with clear guidance regarding re-referral triggers based on PSA levels and changes in urinary symptoms. In the case of patients presenting with urinary tract infections, GPs were asked to treat these patients and review the PSA in 6 weeks with re-referral if required.

### Delphi analysis

A total of two rounds were required to reach a consensus opinion in relation to all the five statements that were circulated to each of the three consultant urologists for consideration. Table 3 shows the initial levels of consensus from the five statements from each round.

In round 1, there was only a 28.3% agreement among the three urologists that the GP referral details were comprehensive and complete (statement 1). For statement 2, there was a complete consensus (100%) that the level of clinical information compiled by the UNP was sufficient to make an effective clinical assessment. Based on these results, statements 1 and 2 did not require further review in round 2 of the Delphi analysis.

Based on round 1 responses, the initial consensus opinion that the management plan was appropriate was 53.3% (32/60), with a discordance of 46.6% (28/60) (statement 3). Statement 4a reached a consensus opinion of 71.6% (43/60). As it had not reached the majority opinion threshold of 75%, this was taken forward to round 2.

The consensus of 73.3% from statement 4b was accepted and the panel, therefore, felt that this should be omitted from round 2, stating that the nursing management plan, compared to a non-specialist urologist would serve as a more relevant end point.

## Round 2

The remaining cases that did not reach consensus in round 1 were included for discussion during a face-to-face meeting with each of the three urologists. All initial responses in round 1 remained anonymous, and the urologists were asked to comment again on the management outcomes of the remaining cases, by an independent moderator.

A revised Likert scale was devised (1 = entirely appropriate; 2 = appropriate, but with minor errors not affecting patient care; 3 = inappropriate with errors potentially significantly affecting patient).

Consensus opinion for statement 3 increased to 87% (52/60 cases), with 10% (6/60) being appropriate, but with minor errors not affecting patient care, and in two cases (3%), the management plan was thought to be inappropriate. In respect of statement 4a, there was an overwhelming consensus of agreement (83.3%) that the management plans outlined by the UNP would not have been done any better by a non-specialist urologist. However, interestingly, in 5/60 (8.3%) cases, the panel’s opinion was that the nurse-led assessment was better than they would have expected from a urology trainee or staff grade doctor.

In round 2, there was discordance in two patient cases (cases 13 and 26) where the assessors felt that the management plan by the nurse was inappropriate with errors potentially and significantly affecting the patient.

### Case number 13

This patient was an inappropriate 2-week-wait referral, presenting with a significant urinary tract infection. The assessors felt the decision to remove the patient from the suspected prostate cancer pathway was appropriate, but the plan to wait for the confirmed results of the urinalysis, in order to prescribe the correct antibiotics until the following day and fax the results to the GP, missed the potential for empirical treatment. The patient was not harmed in this case, but it was felt by the assessors, that there was the potential for worsening of a suspected urinary tract infection which had gone unnoticed by the GP.

### Case number 26

In this case, one urologist felt that management was appropriate and that a non-specialist urologist would have carried out the same treatment. The other two believed that there were minor delays which had the potential to affect patient care. The discussion centred around whether it was appropriate for a 49-year-old with a PSA of 2.5 μg/L to be reviewed again in 4 months rather than having an immediate mpMRI scan, despite a slightly falling PSA, when the PSA remained high for his age, but felt clinically benign on DRE.

The patient in question had originally presented with a PSA 4.3 μg/L (5/5/2016) which on review had dropped to 2.5 μg/L (20/5/2016). Interestingly, however, based on the decision by the nurse to review the PSA in 4 months, the PSA dropped further to 0.8 μg/L (21/9/2016) and no further investigations were subsequently required.

## Cost analysis

Prior to 2012, uroflowmetry provision was not available on the day of assessment for suspected prostate cancer referral; therefore, an additional appointment was made to attend the LUTS clinic; however, the cost of uroflowmetry remained a constant variable. The cost analysis (Table 4) was based on a 30-minute consultation for a new patient appointment by the nurse compared to that of a consultant urologist, staff/Trust grade and registrar with the additional inclusion of 50% on cost tariff for 30 minute of a band 2/3 nursing assistant – £3.44. Within the existing autonomous clinic approach by the nurse, no nursing assistant input was used and, subsequently, no on costs were added. A modest saving was observed compared to any grade of urologist (total cost of UNP versus average cost of urologist + nursing assistant costs) of £20.61/patient per 30-minute appointment. By conducting the uroflowmetry as a one-stop approach and thereby reducing a further follow-up appointment cost of £66, a direct saving to the clinical commissioning groups (CCGs) of £86.61 per patient (average cost of urologist + nursing assistant cost × £66 – UNP cost) was seen.

## Patient-reported outcome measures

Postal questionnaires were sent out to the first 100 patients attending the UNP-led clinic. The response rate was 63% after two mailings. The questionnaire contained 17 questions over three domains which included: referral experience to the hospital, the hospital visit and clinical assessment. Around 57/63 (90%) patients reported being satisfied with seeing a nurse instead of a doctor for their first appointment. About 60/63 (95%) reported that following the initial hospital visit that they had a clear understanding of what the next steps were in their assessment. Overall, 54/63 (86%) were ‘very satisfied’ with the nurse-led service.

## Waiting times

The initial waiting time from GP referral to initial urology assessment was almost halved (52%) across the 3-year period, from 8.4 to an average of 4.4 (total days).

## Cost

Based on our initial 3-year review, there were modest savings, realising a potential saving of £11,500.38 (558 referrals × £20.61/patient). More recent data, however, of the financial year (2017/18), in which the nurse-led clinic saw a significant increase in referrals (*n* = 550), showed a yearly potential saving of £11,335.50. In consideration of staffing cost outside the UK, which is significantly higher than in many countries, greater healthcare saving will be realised. Streamlining the approach as a one-stop clinic for patients will also lead to overall savings in reducing outpatient follow-up costs. It has additionally provided continued flexibility in appointments, thereby reducing unnecessary delays.

## Discussion

In order to ensure that healthcare provision is of the highest quality, safety and value, a highly skilled and experienced multi-professional workforce is needed, if it is to meet the needs of a changing demographic population, healthcare policy and keep abreast of ever-changing healthcare technology [13]. With current shortages in the UK with medical staffing and predictions of a further reduction in the numbers of clinicians by up to 13% by 2020, the projected impact on patient care is likely to be significant. In its 5-year plan, the NHS has clearly outlined that if it is to be fit for the future, it needs to develop new ways of delivering care, including ‘new care models’ that improve productivity and allow ‘integrated care’ and the dismantling of traditional boundaries [14]. Nurse-led services, therefore, have a pivotal role in the future of the NHS and may well be translatable to other healthcare systems.

Autonomous nurse-led clinics are a well-established approach in urological centres in the UK and play a significant role in meeting current population healthcare needs. While there are no directly comparable studies in the published literature for our specific clinical approach, there is an overwhelming consensus from the available literature that nurse-led clinics in urological care are safe, clinically as effective as their physician colleagues and cost-effective [5, 15–21]. This clearly supports the potential benefits of developing such approaches in the UK, with perhaps a wider scope for development in other countries where we believe there is an even greater potential to address more concerning healthcare inequalities through the use and development of nurse-led approaches to patient care, as part of a multi-professional approach [22].

The successful treatment of prostate cancer ultimately, therefore, relies on the detection of the disease at its earliest stages. The ability of nurses to deliver an initial robust cancer-related clinical assessment is, therefore, of paramount importance. Our study found that in only 2/60 (3.33%) of suspected prostate cancer cases reviewed was there a challenge to our clinical approach from all three independent reviewers.

Although the continued advances in cancer care have improved survival and outcomes, the use of patient-reported experience data showing their experiences and understanding of what happens during care processes is being increasingly recognized as important information to improve patient–provider relationships and to evaluate the quality of care delivery [23]. In 57/63 questionnaire responses (90%), patients reported that they had been happy to see a nurse, instead of a doctor for their first appointment; 60/63 (95%) reported that following the initial hospital visit that they had a clear understanding of what the next steps were in their assessment and overall 54/63 (86%) were ‘very satisfied’ with the nurse-led service. Such findings not only demonstrate the acceptance of a nurse-led service but also allow for the best use of clinical resources including manpower, to address and manage the clinical complexities of referrals and provide consistency in service provision through agreed protocols. The evidence supporting a well-informed patient can only lead to an improved patient experience with a positive impact on the quality of patient care, as shown in the National Cancer Experience Survey Programme, 2010 [24].

## Conclusion

Our study demonstrates that a UNP approach to the assessment and management of suspected prostate cancer referrals provides an effective approach to care in an ever-demanding healthcare arena. Through a supported training programme, urology nurses can deliver a high standard of service.

## Conflicts of interest

The authors declare that they have no competing conflicts of interest.

## Funding declaration

The authors declare that they have received no external funding of any sort for this research project.

## Figures and Tables

**Table 1.** Delphi statements.

Delphi statement no.	Statement	Response
1	In your opinion, the GP referral details were comprehensive and complete.	Using a 5-point Likert scale (1 = strongly disagree; 5 = strongly agree).
2	In your opinion, was the level of information from the nurse-led clinic was sufficient to make an effective clinical assessment.	Yes/No.
3	In your opinion, was the management plan by the UNP appropriate.	Using a 3-point Likert scale: (1 = entirely appropriate; 2 = appropriate, but with minor errors; 3 = Inappropriate).
4a	In your opinion, would the management plan for this patient have been done, by a non-specialist urologist.	Using a 3-point Likert scale: (1 = better; 2 = same; 3 = worse).
4b	In your opinion, would the management plan for this patient have been done, by a consultant urologist specialising in prostate cancer.	Using a 3-point Likert scale: (1 = better; 2 = same; 3= worse).

**Table 2.** Treatment approaches following prostate cancer diagnosis (*n* = 150).

Treatment	Number
Active surveillance	52 (33%)
Brachytherapy	6(4%)
Radiotherapy	50 (33.3%)
Surgery (R.A.L.P)	38 (25.3%)
Clinical trials	4(2.7%)

*R.A.L.P – Robotic Assisted Laparoscopic Prostatectomy

**Table 3.** Degree of consensus reached after each round.

Round 1		
Delphi statement no.	Statement	Level of consensus % (*n* = cases)
1	In your opinion, the GP referral details were comprehensive and complete.	28.3% (17/60)
2	In your opinion, the level of information from the nurse-led clinic was sufficient to make an effective clinical assessment.	100% (60/60**)**
3	In your opinion, the management plan by the UNP was appropriate.	53.3% (32/60) Discordance 46.6% (28/60)
4a	In your opinion, would the management plan for this patient have been done, by a non-specialist urologist.	71.6% (43/60) (same) 3.3% (2/60) (better) Discordance 25% (15/60)
4b	In your opinion, would the management plan for this patient have been done, by a consultant urologist specialising in prostate cancer.	73.3% (44/60) (same) 1.6% (1/60) (better) 25% (15/60) discordance

Round 2		
Delphi statement No.	Statement	Level of consensus % (*n* = cases)
3	In your opinion, the management plan by the UNP was appropriate.	86.7% (52/60) 10% (6/60) minor errors Not affecting patient care. 3.3% (2/60) Inappropriate with errors potentially significantly affecting patient care.
4a	In your opinion, would the management plan for this patient have been done, by a non-specialist urologist.	83.3% (50/60) same 8.3% (5/60) better 8.3% (5/60) worse

**Table 4.** Cost analysis for the initial 30-minute clinic appointment.

	Consultant	Staff/Trust Grade Urologist	Registrar	UNP
Salary (30 min)	£40.28	£26.18	£18.81	£11.58
50% healthcare assistant cost	£3.44	£3.44	£3.44	N/A
Total cost/patient/appointment	£43.72	£29.62	£22.25	£11.25
